# 1001 Ways to run AutoDock Vina for virtual screening

**DOI:** 10.1007/s10822-016-9900-9

**Published:** 2016-02-20

**Authors:** Mohammad Mahdi Jaghoori, Boris Bleijlevens, Silvia D. Olabarriaga

**Affiliations:** Department of Clinical Epidemiology, Biostatistics and Bioinformatics, Academic Medical Center, University of Amsterdam, Amsterdam, Netherlands; Department of Medical Biochemistry, Academic Medical Center, University of Amsterdam, Amsterdam, Netherlands

**Keywords:** High-performance computing, Virtual screening, AutoDock Vina, Multi-core, Grid computing, Hadoop, Reproducibility

## Abstract

Large-scale computing technologies have enabled high-throughput virtual screening involving thousands to millions of drug candidates. It is not trivial, however, for biochemical scientists to evaluate the technical alternatives and their implications for running such large experiments. Besides experience with the molecular docking tool itself, the scientist needs to learn how to run it on high-performance computing (HPC) infrastructures, and understand the impact of the choices made. Here, we review such considerations for a specific tool, AutoDock Vina, and use experimental data to illustrate the following points: (1) an additional level of parallelization increases virtual screening throughput on a multi-core machine; (2) capturing of the random seed is not enough (though necessary) for reproducibility on heterogeneous distributed computing systems; (3) the overall time spent on the screening of a ligand library can be improved by analysis of factors affecting execution time per ligand, including number of active torsions, heavy atoms and exhaustiveness. We also illustrate differences among four common HPC infrastructures: grid, Hadoop, small cluster and multi-core (virtual machine on the cloud). Our analysis shows that these platforms are suitable for screening experiments of different sizes. These considerations can guide scientists when choosing the best computing platform and set-up for their future large virtual screening experiments.

**Electronic supplementary material:**

The online version of this article (doi:10.1007/s10822-016-9900-9) contains supplementary material, which is available to authorized users.

## Introduction

Virtual screening (VS) is nowadays a standard step before wet-lab experiments in drug discovery [1, 2]. VS involves calculating the estimated affinities and plausible binding modes of many drug candidates, other drug-like small molecules, or fragments of the former when binding onto a given protein, which is used for short-listing prominent candidates. Even though VS is much cheaper than the lab experiments, it requires investing on the proper High-Performance Computing (HPC) infrastructure in order to enable screening of large ligand libraries. The feasibility of a VS experiment on a given infrastructure can be measured in terms of how long the experiment takes. The longer the calculation time, the less feasible an experiment can become due to practical reasons. Especially, time is directly proportional to cost when performing VS on pay-per-use infrastructures.

The aim of this paper is to provide a concise review together with experimental analysis of the impact of variations in the VS experiment setup and the types of HPC infrastructures on the execution time. This enables a well-informed decision by biochemists when setting up their experiments on HPC platforms. In the experiment set-up, we specifically look at the properties of individual ligands as well as Vina configuration parameters. We use libraries of known and available drugs that are common in biomedical studies. These libraries are also suitable for our purpose because they show a high variety on ligands properties that influence computing time (see “Compound libraries” section).

Currently more than fifty software packages are available for protein-ligand docking, for example AutoDock Vina [3], Glide [4], FlexX [5], GOLD [6], DOCK [7], to name a few. Also, various methods have been developed to speed up their execution [8–12]. We take AutoDock Vina as a typical, arguably most popular, molecular docking tool available for virtual screening. Popularity is explained by being free and the quality of the results, especially for ligands with 8 or more rotatable bonds [13]. Although this paper is based on AutoDock Vina, the findings reported here possibly also apply to similar software packages and analogous types of experiments. For brevity, we will refer to AutoDock Vina simply as Vina in the rest of the paper.

*Paper structure* In the rest of this section, we introduce the basic concepts and features of Vina required for setting up a VS experiment. We then explain the characteristics of the four types of computing infrastructures used to run our experiments. The Methods section presents the ligands and proteins used in the experiments, their various set-ups and configurations, as well as the details of how we ran the experiments on each infrastructure. A complete description of our findings is given in the “Results and discussion” section.

### AutoDock Vina

AutoDock Vina [3] is a well-known tool for protein-ligand docking built in the same research lab as the popular tool AutoDock 4 [14, 15]. It implements an efficient optimization algorithm based on a new scoring function for estimating protein-ligand affinity and a new search algorithm for predicting the plausible binding modes. Additionally, it can run calculations in parallel using multiple cores on one machine in order to speed up the computation. In this paper, we adopt the following terminology (the italicized terms).

One *execution* of Vina tries to predict where and how a putative ligand can best bind to a given protein, in which Vina may repeat the calculations several times with different randomizations (the configuration parameter *exhaustiveness* controls how many times to repeat the calculations). The part of the protein surface where the tool attempts the binding is specified by the coordinates of a cuboid, to which we refer as the *docking box*. This is called the “search space” in the Vina manual.

By default, redoing the same execution on the same ligand-protein pair can produce varying binding modes because of the randomized seeding of the calculations. Nevertheless, Vina allows the user to explicitly specify an initial randomization *seed*, so that the docking results can be reproduced.

Since the repeated calculations in one execution are independent, Vina can perform them in parallel on a multi-core machine. To do so, it creates multiple *threads*: the threads inside a program will run in parallel whenever the cores are free. The maximum number of simultaneous threads can be controlled when starting the docking experiment (using command-line option *cpu*). By default, Vina tries to create as many threads as the number of available cores.

### Infrastructures

High-performance computing infrastructures with several levels of computational capacity are typically available to researchers today. In the simplest case, one can take advantage of personal computer’s multiple cores to speed up, or *scale* an experiment. Nowadays, small and medium research groups and enterprises can afford compelling computers with tens of cores. Another alternative is to use accelerators, i.e., hardware that can be used next to the central processor (CPU) to accelerate computations. Examples are graphical processing units (GPU) and Intel’s recent development called Xeon Phi, which can have hundreds of (special-purpose) processing cores. In the extreme case, supercomputers with millions of cores can be used. It is however very economical to make a network of “ordinary” computers and use a so-called *middleware* to distribute the jobs among the available computing cores. This is called distributed computing. We use the term *cluster* to refer to a network of computers that are geographically in the same place, and the term *grid* for a network of geographically scattered computers and clusters. A widely used middleware for clusters is called portable batch system (PBS), which is capable of queuing incoming jobs and running them one after the other. A more advanced middleware is Hadoop [16], which has efficient file management and automatically retries failed or stalled jobs, thus greatly improving the overall success rate. Finally, grids may constitute dedicated resources (e.g., using gLite [17] middleware) or volunteered personal computers connected via internet (e.g., BOINC [18]).

*Cloud* The main idea of cloud computing is to use virtualized systems. It means that organizations do not have to invest upfront to buy and maintain expensive hardware. Instead, they can use hardware (or software services running on that hardware) that is maintained by the cloud providers. It is possible to use a single virtual machine or create a cluster on the cloud. Hadoop clusters are usually among the standard services offered by commercial cloud providers. With these pay-as-you-go services, cloud users pay only whenever they use the services and for the duration of use. Although in this paper we used physical grid and cluster infrastructures, the results are generally applicable also to analogous virtual infrastructures.

In this study, we only use national resources that are available for free to academic researchers in The Netherlands, which are maintained by the nationally funded SURF organization.^1^ These resources include four different types of infrastructures that are representative of alternatives typically available to scientists worldwide. Table 1 summarizes the characteristics and capacities of these systems. The smallest of all is an 8-core virtual machine on the Dutch Academic HPC cloud. The second is a local cluster at the Academic Medical Center of the University of Amsterdam (AMC) with 128 cores and PBS middleware. The third infrastructure considered here is the Hadoop cluster for scientific research operated by SURFsara. This cluster consists of 90 data/compute nodes (adding up to more than 1400 cores) and has a distributed file system with a capacity of 630 TB. Finally, we use the Dutch eScience grid which includes a dozen PBS clusters all around the country (including ours). The Dutch grid uses the gLite middleware [17].

**Table 1:** Characteristics of the infrastructures used in the experiments

	Total cores	CPU speed (GHz)		Memory per core (GB)	CPU types
		Min	Max		
Single machine (on HPC cloud)	8	2.13		1	Intel Xeon
AMC local cluster	128	2.3		4	AMD Opteron
Dutch Academic Hadoop cluster	1464	1.9	2.0	≥6	Intel Xeon - AMD Opteron
Dutch Academic grid	>10,000	2.2	2.6	≥4	Intel Xeon - AMD Opteron

*Note* Because the hardware characteristics of the exploited infrastructures are very diverse (see Table 1), it is unfair to directly compare them based on the execution times. The usage of these infrastructures in our experiments is meant as illustration for a variety of case studies, and not as a benchmark.

## Methods

### Virtual screening set-up

The examples and results presented in this paper are based on virtual screenings for two target proteins and four well-known ligand libraries. These experiments were run as part of collaborative efforts with AMC biomedical scientists. Various combinations are used to create case studies that are controlled, but at the same time realistic to illustrate different ways to run Vina in various infrastructures.

#### Proteins

Target proteins were selected based on current research interests in the department of Medical Biochemistry at the AMC.

- Alpha-ketoglutarate-dependent dioxygenase FTO [19] is a protein implicated in the development of obesity. FTO is the strongest genetic predictor of increased body weight [20]. It uses alpha-ketoglutarate as a cofactor to remove methyl groups from mammalian mRNA. Substrate binding occurs close to the catalytic site. To discover potential inhibitors for FTO docking was performed on the crystal structure 3LFM^2^ [21] using a box size of $32 \times 32 \times 32 = 32{,}768\,\AA ^{3}$, centered around the enzyme’s active site.
- NUR77 [22]: NUR77 is a nuclear receptor implicated in vascular remodeling and atherosclerosis. It is classified as an orphan receptor, as up to now no activating ligand has been identified. In an attempt to identify potential binding sites, NUR77 virtual screens were run on crystal structure 3V3E^3^ [23] against two docking box sizes: the complete ligand binding domain (LBD) surface (Big box) and against a small part of the LBD surface (Small box). The exact box sizes are:
  - Small box: $$18 \times 18 \times 18 = 5832\,\AA ^{3}$$.
  - Big box: $$66 \times 56 \times 60 = 221{,}760\,\AA ^{3}$$.

PDB structure files of target proteins were downloaded from the RCSB repository and the protein structures were analyzed with PyMOL.^4^ In case of multiple molecules in the unit cell (oligomers or hetero-complexes), the target molecule was isolated either by using the save molecule option in PyMOL or by directly modifying the PDB file. Polar hydrogen atoms were added to the selected structure and a .pdbqt receptor file was created using AutoDockTools^5^ [14].

#### Compound libraries

The ZINC (ZINC Is Not Commercial) [24] database contains over 20 million chemical compounds, about two thirds of which are commercially available. Actually, ZINC contains compound models where a compound may be represented several times with its different enantiomers and protonation states in separate models. We use the terms ‘compound’ or ‘ligand’ to refer to these models. We selected a number of sub-sets from the ZINC database varying in size from a few dozens to up to almost 100K compounds. These sub-sets (compound libraries) also represent diverse types of molecules as explained below. The libraries were downloaded directly in the .pdbqt format that is required by Vina.^6^

- Nutraceuticals (Nutra) [25]: A small library of 78 compounds from the Drugbank^7^ containing, amongst others, diet supplements, often with therapeutic indication.
- Human Metabolite Database (HMDB) [26]: This library with 2,462 compounds contains information about small molecule metabolites found in the human body.^8^
- FDA Approved Drugs (FDA): Database of 3358 commercially available drugs that are approved by the FDA (US Food and Drug Administration) with acceptable safety/toxicity for medical treatments.
- Zinc Natural Products (ZNP): This is the biggest library considered in our experiments, with 89,398 compounds, comprising nature-inspired drug-like compounds.

These libraries are a logical choice for biomedical scientists in their virtual screening experiments, because they contain known and available drugs—see for example these case [27–29]. This approach has the advantage of avoiding long drug development, making it possible to move quickly into more advanced phases of drug testing. Note that these libraries, however, differ from datasets like DUD-E [30] or Dekois [31], which are synthetic benchmarks suitable for evaluating the quality of docking algorithms. Benchmarking is not the goal of this study; the experiments here are aimed only at illustrating characteristics of this docking tool under heavy workload in the scope of high performance computing. Furthermore, the drug compounds included in the abovementioned libraries display large variation in their characteristics. Specifically, we are interested in the number of active torsions (rotatable bonds) and the number of heavy (i.e., not hydrogen) atoms in the ligands. These are important factors affecting the docking process, therefore expected to affect also the execution time of Vina.

**Fig. 1:**
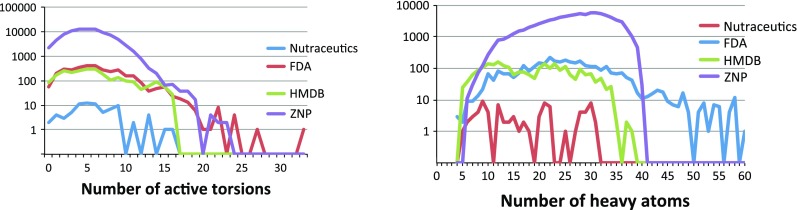
Distribution of ligands in each library (showing the counts in logarithmic scale) based on the number of active torsions (*left*) and heavy atoms (*right*)

The compounds in these four libraries add up to a total of 94,649 molecules (considering the duplicates in different libraries). Figure 1 (left) shows the count of compounds when grouped based on their number of active torsions. In all four libraries considered, 7,082 compounds (7.43 %) have 0 or 1 active torsions and only 6,872 (7.21 %) have 10 or more. In practice, however, compounds with too many rotatable bonds could be excluded from VS (for example using tools like Raccoon2^9^), since such compounds are not expected to produce accurate results with existing docking tools. Figure 1 (right) shows the distribution of the compounds based on how many heavy atoms they have. We see again that except for some compounds in FDA (a total of 151), the others are composed of no more than 40 heavy atoms.

### Implementations

The simplest way to screen a ligand library is to run Vina on a multi-core machine, but this is suitable only for smaller libraries. For this purpose, many researchers use the readily available scripts, such as those provided by Vina authors,^10^ which automatically process all the ligands in a library one after the other, or Raccoon2 or PyRx.^11^ However, the results in “Results and discussion” section indicate that these scripts can be improved. To scale up to bigger libraries, one may use a cluster or grid. Whenever more processing cores are available, higher speed-up is expected, but in practice there are many other determining factors such as the the balance between overhead vs. efficiency of distributing the jobs and collecting the results, the chance of job failure and the ability of the system to recover (fault tolerance). Below we explain how we performed VS on the different infrastructures focusing on high-level considerations that may affect the execution time.

#### Multi-core

**Fig. 2:**
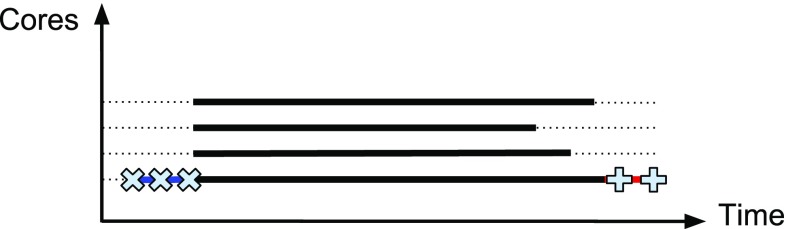
Schematic diagram of core occupancy along time for a single execution of Vina on four cores. Each *line* represents one core. The preparation steps (marked *times symbol*) and post-processing (marked *plus symbol*) are performed on one core. The actual docking process (*bold black*) is parallelized. The *dotted lines* show when the cores are idle

**Fig. 3:**
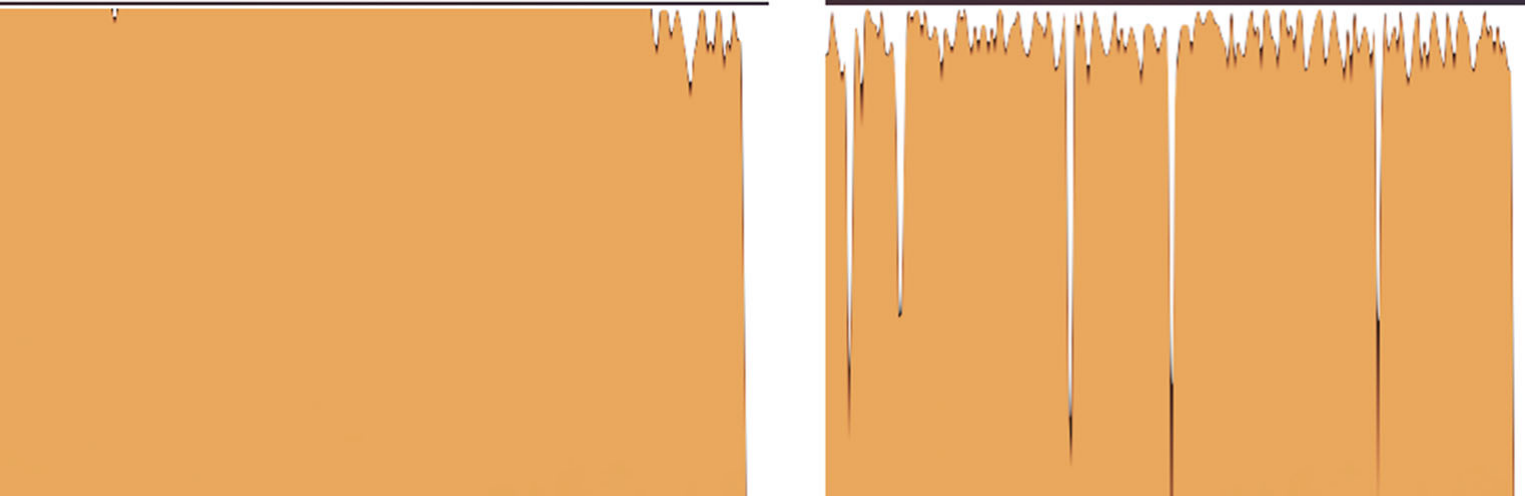
CPU usage, captured by the CPU monitoring tool XRG, showing the cumulative load on four cores during virtual screening. On the *left*, at every moment two ligands are processed in parallel, while on the *right*, standard scripts provided on the Vina website are used where ligands are processed one at a time. The *right figure* shows a visible fall on CPU load when switching to the next ligand as predicted in Fig. 2

In processing one ligand, Vina has the ability to take advantage of multiple cores available and perform the calculations in parallel (called internal parallelism). However, every execution includes pre- and post-processing steps which are not run in parallel, e.g., pre-calculating grid maps describing the target protein or reading/writing files. Even though very short, these sequential steps cause some cores to be idle as illustrated in Figs. 2 and 3, which will increasingly delay the calculations when screening thousands of ligands.

In a virtual screening experiment, multiple ligands are considered, and can therefore be processed in parallel (called external parallelism). We used a *process pool* in the Python language to start a fixed number of *N* concurrent processes on a multi-core machine, each of which running one instance of Vina. When a Vina instance finishes, the corresponding process picks up the next outstanding ligand. Figure 3 shows CPU usage graph (made by the XRG tool^12^) for docking one ligand vs. two ligands at a time.

Suppose *N* ligands are processed at a time (external parallelism level), and Vina is configured to create *M* threads in parallel for each ligand via the *cpu* option (internal parallelism level), the total parallelism level is then given by $N*M$. We carried out several tests on an 8-core machine to see which combinations of internal and external parallelism produce the best speed-up. We considered all combinations such that $8 \le N*M \le 32$, because these fully exploit the 8-core capacity without overloading it. The Python scripts to run the experiments on a multi-core machine are provided as supplemental material.

#### Hadoop cluster

Hadoop [16] is an open-source implementation of the map-reduce paradigm, originally introduced by Google for parallel processing of many small data items. In this paradigm, first parallel instances of a *mapper* job process the input items, producing a series of key-value pairs. The system sorts these pairs by their keys and passes them on to the *reducer* jobs that will aggregate the outputs. In VS, each ligand corresponds to one input, thus creating one mapper job per ligand that runs an instance of Vina. These jobs output the binding affinities as keys together with the name of the ligand as the value. Therefore, the binding affinities are automatically sorted by the system. One reducer job is enough for collecting all the outputs and the sorted affinities.

#### Local cluster and grid

**Fig. 4:**
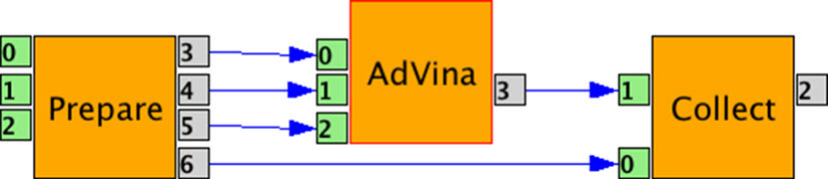
WS-PGRADE workflow for distributed execution of Vina and collecting the output results on local cluster and grid (see text for explanation)

Our first implementation for cluster and grid infrastructures uses the WS-PGRADE workflow management system [32]. The workflow is illustrated in Fig. 4, which can be built from a graphical user interface. This approach enables running the virtual screening and collecting its results as explained below. WS-PGRADE can run this workflow both on the AMC local cluster and on the Dutch grid.

When using a cluster or grid, some overhead is introduced to manage the jobs. This disfavors the execution of many small jobs, as compared to Hadoop and multi-core infrastructures. To reduce the overhead, a smaller number of bigger jobs should be created. To do so, more than one ligand should be put into each compute job [9, 10, 33]. Based on this idea, the three components of the workflow in Fig. 4 implement respectively the following three steps:

- Prepare: splits the input library into several disjoint groups of ligands to be processed in parallel.
- AdVina: runs Vina on these groups of ligands in parallel.
- Collect: merges all the outputs and sorts the ligands based on their binding affinity.

For running on the grid, we made a second implementation using the DIRAC pilot-job framework [34], which allows for better exploitation of the resources. Pilot jobs enable us to take advantage of less busy clusters and therefore avoid long queuing times. Additionally, one pilot job can run various instances of Vina jobs without introducing middleware overhead, therefore increasing efficiency.

For the grid experiments in this paper, we used the AMC Docking Gateway^13^ [9] with the DIRAC middleware. This gateway allows for specifying the basic configurations: docking box coordinates, exhaustiveness, number of modes and energy range. The distribution on the grid and collection of the results are automated, as well as sorting the outcomes based on the highest binding affinity. Additionally, it allows the user to download partially completed results [35] and it provides provenance of previous experiments.

### Summary of VS experiments

Considering the three possible docking boxes (FTO, NUR77 Small and NUR77 Big) and the four ligand libraries (Nutra, HMDB, FDA and ZNP), there are a total of 12 cases for virtual screening experiments that have different computational demands. Table 2 summarizes the combinations that were tried on the considered infrastructures. Some cases were repeated with different configurations by varying exhaustiveness, seed, or number of threads.

**Table 2:** This table shows the four ligand libraries, four infrastructures, and the three docking boxes (FTO and the big and small boxes on NUR77)

	Multi-core	AMC cluster	Hadoop cluster	Grid
Nutra	Small	Small	Small	Small
	Big	Big	Big	Big
	FTO	FTO	FTO	FTO
HMDB	Small	Small	Small	Small
	Big	Big	Big	Big
	FTO	FTO	FTO	FTO
FDA	Small	Small	Small	Small
	Big	Big	Big	Big
	FTO	FTO	FTO	FTO
ZNP	Small	Small	Small	Small
	Big	Big	Big	Big
	FTO	FTO	FTO	FTO

When a name “Small”, “Big” or “FTO” is in black, it shows that we ran VS experiments with that docking box on the corresponding infrastructure and ligand libraries

On multi-core, we ran the Nutra library a total of 59 times with different parallelism levels. Note that screening of ZNP library on an 8-core machine is not feasible, as it would need almost one year to complete. On our local cluster, we ran all the libraries and with different configurations, but due to its relatively small capacity we did not try all cases. On the bigger platforms, namely Hadoop and grid, we tried almost all cases.

Whenever we needed comparison of execution times, we used the experiments executed on the local cluster, which has a homogeneous platform (hardware and software stack). The analyses were based on average execution times of repeated runs.

## Results and discussion

The results in each of the following subsections are based on various experiments in Table 2, but we select one of them in each section in order to exemplify the reported findings. The graphs are therefore shown only for one case in each subsection.

### Internal versus external parallelization

**Fig. 5:**
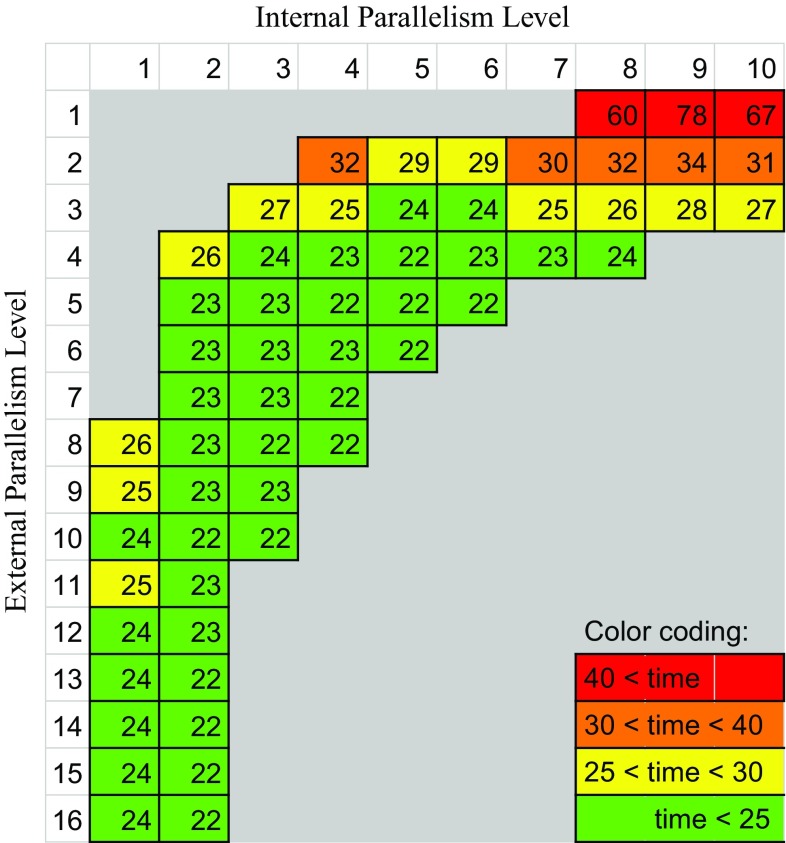
Execution time (in min) for combinations of internal and external parallelism on multi-core. *Columns* correspond to the level of internal parallelism (*M* = 1–10) and *rows* correspond to external parallelism (*N* = 1–16, i.e., up to twice the number of cores available). *Empty boxes* represent cases where the system is underutilized because some cores are left idle ($N*M < 8$), or overloaded ($N*M > 32$). *Color* coding: *green* for fastest runs, changing to *yellow* and finally to *red* for slowest runs

Figure 5 shows the execution times for 59 combinations of internal and external parallelism. In these measurements on this specific setup and hardware, we observe that the fastest execution time with the smallest load on the system corresponds to the parallelism level 20, i.e., with internal and external parallelism of 4 and 5. This is clearly above twice the number of available cores, indicating that system saturation is beneficial in this case.

Based on these specific measurements, we cannot give a golden formula for the best combination of internal and external parallelism, as it may depend on various factors ranging from hardware characteristics to configuration parameters like exhaustiveness. Nevertheless, since we also observe that increasing internal parallelism at some point reduces the performance, we can conclude that the optimal solution is obtained by balancing internal and external parallelism. We see that this strategy, compared to pure internal parallelization offered by Vina (first row in Fig. 5), leads to at least a twofold speed-up. When running on cloud resources, this speedup would translate to lower costs, since less time/core can be used for running the VS.

### Reproducibility of results

Vina calculations are based on pseudo-random generation. Such programs, if provided with the same initialization—called seed, produce the same behavior. By varying the random seed, different docking results can be generated, thus allowing the user to select the best results.

**Fig. 6:**
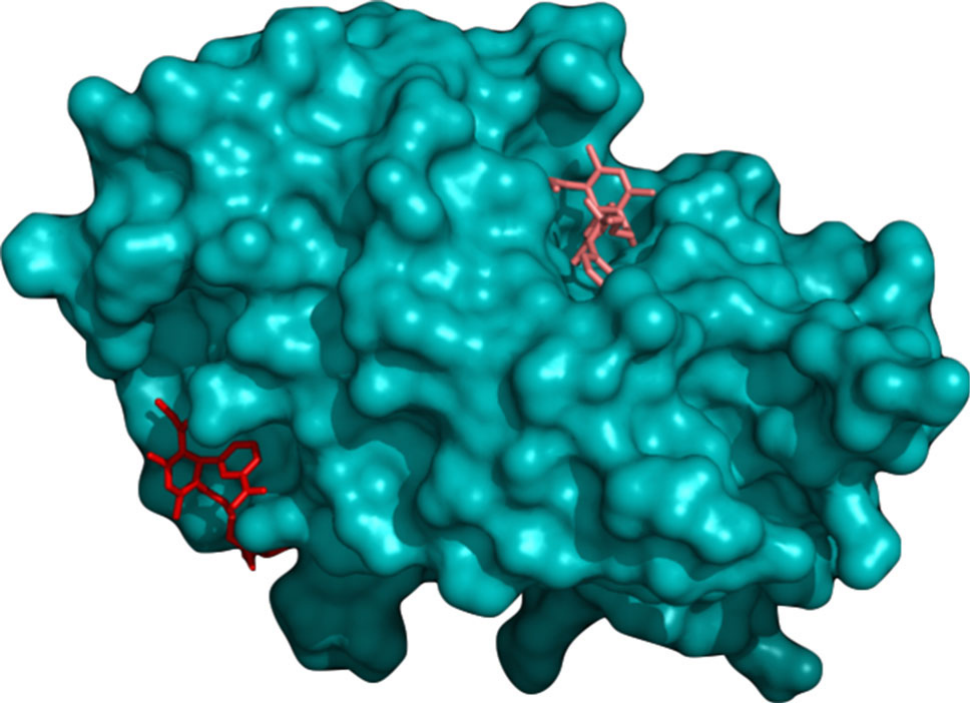
Two binding modes for the same ligand on NUR77 reported by Vina with the same configuration, including the randomization seed, but run on different platforms

We observed that different operating systems, e.g. Mac vs. Linux, or even different versions of CentOS (a common Linux-based system), generated different outcomes, even when Vina was run with the same randomization seed and input parameters. For example, Fig. 6 shows two binding modes reported for the same ligand on the surface of NUR77, using identical seeds but run on different systems. Since docking studies were performed on an isolated monomer, both docking poses are definitely different. The calculated energies for these two binding modes differ by 0.7 kcal/mol. This shows a lack of accuracy in the Vina manual, which states that “exact reproducibility can be assured by supplying the same random seed to both calculations, but only if all other inputs and parameters are the same as well.”

For reproducibility, one needs to make sure to record the characteristics of the used platform together with the random seed and other configuration parameters. A similar phenomenon has been reported for DOCK in [36], where the authors suggest to use virtualized cloud resources to overcome this issue. Nevertheless, reproducibility of the results may be completely endangered in the long term because the exact same platforms may not exist anymore. For example versions of an operating system are usually discontinued after at most a decade.

### Impact of docking box size

Depending on prior knowledge about the target, the screening can be constrained to a limited area instead of the total protein surface, by specifying a docking box. In this case, one may naively expect that the computation time is reduced.

**Fig. 7:**
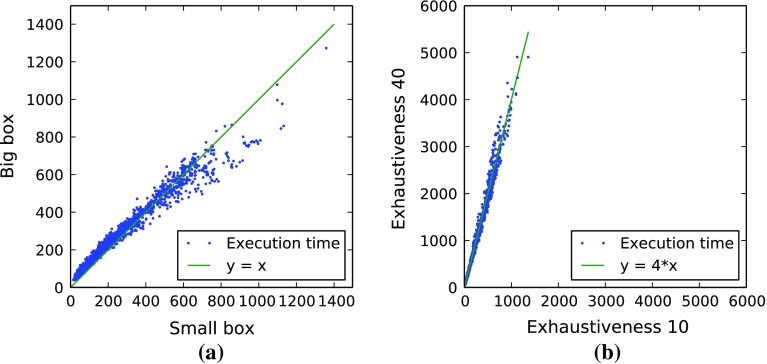
Comparison of execution times (in s) for different Vina configurations. Every *blue dot* in the plots represents one ligand. **a** NUR77 Small box versus Big box (HMDB, exhaustiveness = 10). **b** Exhaustiveness 40 versus 10 (HMDB, NUR77 small box)

Figure 7a compares the execution time of screening HMDB against NUR77 with exhaustiveness 10 and a fixed randomization seed on the homogeneous AMC cluster. The results depicted here show that execution time is not consistently larger or smaller for either Big or Small box. The same result was obtained for similar experiments we conducted with exhaustiveness 40 on HMDB and with exhaustiveness 10 on FDA. Keep in mind that the Big box is more than 38 times larger in volume than the Small box. We therefore conclude that the time required for every run is not entirely dependent on the total size of the docking box. In other words, enlarging the docking box does not necessarily mean that Vina is going to spend more time on the calculations. In order to ensure good quality in the docking results, one needs to enforce more runs. This is suggested in the Vina manual, which states that when the docking box volume is above $27{,}000\,\AA ^{3}$ (as is the case for the Big box), “the search algorithm’s job may be harder. You may need to increase the value of the exhaustiveness to make up for it”.

A recent study [37] proposes an algorithm to determine the optimal box size when a specific pocket is targeted, such that the quality of Vina calculations is higher. As we have shown, when using such optimizations, one does not need to worry about any (consistent) change in execution time.

### Impact of exhaustiveness

In the various settings used for comparison, we see an almost linear increase in the execution time when increasing exhaustiveness. In Fig. 7b we show the execution times for the case of screening HMDB ligands against the Small box of NUR77 in the two settings of exhaustiveness 10 and 40 with a fixed randomization seed on the homogeneous AMC cluster.

Our results confirm the Vina manual, which states that increasing the exhaustiveness “should increase the time linearly”. The manual suggests that higher exhaustiveness “decreases the probability of not finding the minimum [binding energy] exponentially” because more runs are performed, however all using the same seed. One may alternatively perform an equal number of runs by executing Vina repetitively, but with a smaller exhaustiveness each time. This will take the same amount of total time (as we have seen here), but since various randomization seeds can be used in each Vina run, there could be more variety in the results with a better chance of finding the minimum binding energy.

### Impact of ligand properties

**Fig. 8:**
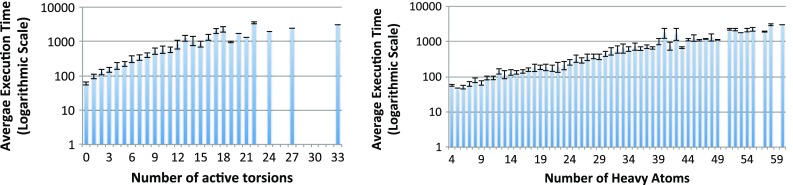
Average execution time (in s) for ligands grouped by number of active torsions (*left*) and heavy atoms (*right*). Results are for screening FDA against complete surface of NUR77 with exhaustiveness 10 on local cluster. *Bars* represent mean execution time with standard deviation as *error bars*

The Vina authors already show a relationship between execution time and ligand properties based on a small experiment with 190 protein-ligand complexes [3]. Here we repeat the experiment for a much larger number of ligands. The graphs in Fig. 8 show execution time in logarithmic scale for screening FDA against complete NUR77 surface with exhaustiveness 10, with results grouped into bins of same number of active torsions or heavy atoms. We chose FDA because it has a larger variation in both number of active torsions and heavy atoms (see Fig. 1). Note that the computation time can vary significantly for different ligands, from 35 s to 65 min in the case of this target and library. However, on average the execution time grows proportionally to the number of active torsions (and similarly, heavy atoms), with a few outliers. Although deriving the form of this relation, i.e., whether execution time grows exponentially or polynomially, requires further statistical analysis, we can nevertheless benefit from this relation in optimizing overall execution time a VS experiment, as elaborated below.

First consider systems like a grid, where ligands are grouped to create bigger compute jobs (cf. “Methods” section). Kreuger et al. [33] have studied the number of ligands in each group for optimal effiency. But if a group happens to contain many large or flexible ligands, it will have a much longer execution time that will dominate the execution time of the whole VS experiment. Zhang et al. [38] propose a preprocessing step on the ligands to find their similarities as a basis of balancing the groups. Using number of active torsions and heavy atoms requires much less computational effort, therefore making it easier to adopt for new ligand libraries. Nevertheless, the effectiveness of this approach for predicting execution time of each ligand group remains to be studied.

**Fig. 9:**
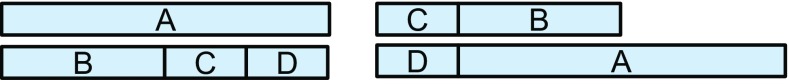
The effect of load balancing by processing larger and flexible ligands first on total virtual screening time

In other systems that handle ligands one by one (for example on a multi-core machine or a supercomputer), we recommend to start the VS experiment by first processing large and flexible ligands. By leaving smaller ligands that take less time for a later stage, an automatic load balancing between the processing cores happens, as illustrated in Fig. 9. Here we show four ligands that are processed on two cores. On the left scenario, larger ligands A and B are processed first. Since A takes much longer than B, both C and D will be processed on the same core. But if we had started with the smaller ligands C and D in parallel (on the right), we would end up running A and B also in parallel, which in the end results in one core being idle while A is still being processed. Clearly the scenario on the left has a faster overall execution time as it can better utilize the available cores. A similar method is used by Ellingson et al. [39], although in their work only the number of active torsions were considered and not the number of heavy atoms.

### Choice of infrastructure

We summarize in Table 3 the execution time for various virtual screening experiments on four different infrastructures. In these experiments, Vina is configured to use one processing core only (i.e., turning off the multi-core feature). Total cores shows the compute capacity of each infrastructure, which is proportional to the size of virtual screening experiment (number of ligands to screen). On multi-core and Hadoop, the docking of each ligand defines one compute job, whereas for the AMC cluster and grid, we make compute jobs by grouping ligands to reduce overhead (see “Methods” section for more explanation). ‘Max Parallelism’ shows the maximum number of ligands that were being processed at the same time. On the multi-core machine, this is equal to the external parallelism level. In other cases, this is equal to the maximum number of compute jobs that were running at the same time, and would ideally get as close to the total number of cores as possible. ‘Avg parallelism’ is calculated by taking the average of the parallelism level in every second over the whole experiment time span (disregarding idle times), and as such shows how effectively the infrastructure has been utilized in each experiment. The actual level of parallelism that can be achieved is hampered by various factors as explained below.

**Table 3:** A summary of execution time and achieved parallelism on various infrastructures and middlewares. See text for details

	Total cores	# Ligands	# Compute jobs	Max parallelism	Avg parallelism	Total wall clock	Avg time per ligand (sec)	Fault tolerance	Chance of failure
Multi-core	8	78	78	8	6.7	0:26:13	20.167	Very low	Very low
				10	8.7	0:24:40	18.974		
				12	10.4	0:24:20	18.718		
		3358	3358	8	7.4	27:11:10	29.145		
AMC cluster (WS-PGRADE)	128	2462	164	14	7.3	34:16:03	50.107	Low	Medium
				76	34.6	4:49:32	7.056		
				95	37.9	4:21:33	6.374		
		3358	223	53	11.4	75:48:44	81.276		
				124	28.9	12:18:07	13.189		
		89,398	1004	128	62.0	320:50:22	12.920		
Hadoop cluster	1464	89,398	89,398	1384	824.9	8:37:13	0.347	High	Medium
				1307	761.6	9:56:40	0.400		
				1361	114.4	54:46:08	2.206		
Grid (DIRAC)	>10,000	89,398	1116	1080	169.8	23:23:22	0.942	Medium (DIRAC)	Very high
				1070	165.2	25:34:41	1.030		
			5578	1370	593.7	6:41:02	0.269		

The studied infrastructures comprise shared resources (except for the multi-core case), which means we may get only a part of its capacity if other users are running their experiments at the same time. This is very clearly seen in the experiments run on the AMC cluster, where the maximum parallelism is in most cases much lower than the number of available cores. In the case of grid, this is not visible due to its very high capacity, and therefore, given a fixed number of compute jobs one could truly expect a more or less fixed level of parallelism.

On a multi-core computer the chance of the machine failing is very low. But when connecting some computers (in a cluster or grid), there is a higher chance that at least one of them fails. Additionally, other factors like physical network failure or access to remote data mean that, as the number of connected computers grows, the chance of failure grows much faster. Fault tolerance in this context can be simplistically defined as the ability to automatically restart a docking job whenever the original run fails. Such failures penalize average parallelism, especially if they are manually retried, as can be seen for example in the experiments on the AMC cluster.

From the analysis of Table 3 the following conclusions can be drawn. Small screening experiments (e.g., using Nutra library) can be easily performed on a multi-core machine. Slightly bigger experiments can be done faster with bigger number of cores. We see that for a few thousand ligands (like HMDB or FDA), the results are available in a matter of a day with an eight core machine. A small cluster can be a reasonable choice for such medium-sized experiments. Nevertheless, we see that failures and manual retries may gravely increase the perceived execution time (wall clock). Hadoop’s high fault tolerance mechanisms offer a good solution, as it has the smallest difference between max and average parallelism. From our experiments, Hadoop seems to be the best option when no competition exists for the resources; otherwise, execution time may be still affected by interference of other users’ jobs on a shared infrastructure. Grid is based on resource sharing, with a stable ratio between average parallelism and number of compute jobs. But the low ratio between max and average parallelism show the great deal of overhead and competition for resources on this type of infrastructure.

*Virtualization* Typical usage of cloud involves allocating virtual machines (VM), where it is possible to define—and pay—for the number of cores, amount of memory and disk size. Since Vina does not require much memory (roughly in the order of maximum 100MB per run/thread), one can invest the available budget on more cores. Most commercial cloud providers nowadays also offer the possibility of deploying a Hadoop cluster on virtual resources. This is a more expensive resource though. Given the importance of handling failures when large VS are performed, it would be interesting to investigate whether the fault-tolerance facilities of Hadoop might compensate for its extra cost on the cloud.

*Other infrastructures* A supercomputer, if at disposal, is a suitable platform for large VS experiments. By means of supercomputers with thousands of cores, one million compounds were screened using Vina in 24 h by Ellingson et al. [40], and in 1.4 h by Zhang et al. [41]. A powerful alternative is a grid of volunteer resources, like the World Community Grid composed of over 2 million personal computers connected via internet. For example, the GO Fight Against Malaria project on World Community Grid performed over 1 billion different Vina jobs in under 19 months [42]. For not such large experiments, a good alternative to a multi-core CPU is to use accelerators. However, this requires rewriting the docking software; therefore, we did not consider it for Vina. Two options, GPU and Xeon Phi, have been shown to be suitable for parts of the docking process as described in [43]. Around 60 times speed up is obtained with their in-house developed virtual screening software.

## Conclusions

Vina has the ability to perform molecular docking calculations in parallel on a multi-core machine. We found out, however, that Vina does not exploit the full computing capacity of a multi-core system, because some pre- and post-processing needs to be performed using only one core. Therefore, external parallelization approaches should be employed for increasing the efficiency of computing resources usage for VS. In our experiments, this led to more than a twofold speed-up.

We also found that the use of the same randomization seed does not always assure reproducibility. In fact, docking results are reproducible only if performed on the exact same platform (operating system, etc.). We observed in some cases that the same seed and calculation parameters can lead to diverging results when used on different platforms: both different binding modes and energies were reported.

Further study on the execution time confirmed previous knowledge about Vina, but on a much larger dataset: execution time is linearly proportional to exhaustiveness (number of simulations per run). It is therefore advisable to run Vina several times rather than increasing the exhaustiveness. It takes as long and at the same time, and in this way multiple seeds are taken, perhaps elevating the chances of getting closer to the best binding mode.

We also saw that execution time increases with the number of active torsions and heavy atoms in the ligands. This is in line with the Vina manual statement that the number of steps in one run is “determined heuristically, depending on the size and flexibility of the ligand” among others. This heuristic function could be useful to improve load balancing, for example when ligands are grouped for execution on the grid, or to order the execution on multi-core or supercomputers.

A counter-intuitive finding of our study is that we observed no relation between the size of the docking box and execution time. Our observations merit further exploration into this issue. It is also of interest to investigate whether the quality of the results is comparable when different sizes of the protein surface are covered. Since a very low exhaustiveness value might lead to premature results, such a study on the quality of the results must ensure a suitable exhaustiveness.

Enabling biochemical scientists to perform large VS experiments is not solely about improving execution time. Even though we did not consider them all in this paper, these are topics of continuous research. We did not elaborate on the efforts needed to port Vina to any of the infrastructures, because this can be a one-time investment. Ideally, all the details of translating a VS experiment to compute jobs on the underlying infrastructure, and collecting and aggregating the outputs can be hidden under the hood of a dedicated gateway with a simple to use interface [9, 44]. Such gateways can additionally help the researchers with management of the large amount of inputs and outputs, including provenance and post-processing. Analysis and interpreting these big amounts of data is still a young research topic.

## Electronic supplementary material

Supplementary material 1 (py 1 KB)

## Footnotes

1 http://surf.nl.

2 http://www.rcsb.org/pdb/explore/explore.do?structureId=3LFM.

3 http://www.rcsb.org/pdb/explore/explore.do?structureId=3V3E.

4 http://www.pymol.org.

5 http://autodock.scripps.edu.

6 http://zinc.docking.org/pdbqt.

7 http://www.drugbank.ca.

8 http://www.hmdb.ca.

9 http://autodock.scripps.edu/resources/raccoon2.

10 http://vina.scripps.edu/vina_screen_local.sh.

11 http://pyrx.sourceforge.net/.

12 http://www.gauchosoft.com/Products/XRG/.

13 http://docking.ebioscience.amc.nl/.
